# Dr. DeWilton Snowden

**Published:** 1897-12

**Authors:** 


### Obituary.

### DR. DE WILTON SNOWDEN DEAD.

**He Passes Away at Laurel at the Age of Eighty Years. A Well-Known Physician. Among the First Marylanders to Espouse the Cause of the South. Connected with Some af the Old Families of the State and Brother of the Late Henry Snowden. A Member of the House of Delegates in 1880. Sketch of His Interesting Career.**

Dr. De Wilton Snowden, one of the oldest citizens of Laurel, died at midnight Wednesday, November 25th, at the home of his son-in-law, Dr. Emory G. Valk, on Washington avenue, Laurel, Md. He had been in feeble health for a year, suffering from a disease of the heart. Last winter he was dangerously ill for a long time, but during the summer he was able to walk about town and attend to his business affairs as usual. He was out of doors for the last time on Tuesday. On the same day he was again taken ill, and Dr. John D. Cronmiller, his attending physician, was summoned. He exerted his skill to revive the flagging energies of his patient, but Dr. Snowden was beyond the aid of science. He grew weaker, although retaining consciousness most of the time, until the end came, peacefully, in the presence of several members of his family.

Dr. Snowden was eighty years of age. He was born at "Montpelier," three miles from Laurel, the homestead of his branch of the Snowden family. He was the third son of Nicholas Snowden, and his wife, whose maiden name was Warfield. Nicholas Snowden died about 1840. He had six sons, Thomas, Edward, De Wilton, Henry, Arthur and Nicholas. Of the five daughters, only one survives—Miss Eliza Snowden—who is now a nun in a convent at Georgetown, D. C. The eldest daughter married Mr. Frank Hall, and afterwards, Mr. Charles Hill; another became the wife of Mr.

Charles C. Hill, and the mother of Mr. F. Snowden Hill, ex-collector of internal revenue for the Maryland district. The others were Mrs. Capron, wife of Colonel Horace Capron, and who was influential in the establishment of St. Philip's Church; Mrs. Jenkins, wife of Dr. Theodore Jenkins; Mrs. Bowie, wife of Colonel Walter W. W. Bowie.

Dr. Snowden was a great-great-grand son of Richard Snowden, who came to Maryland from Birmingham, England, in the seventeenth century, and secured a patent from the Crown for "Birmingham Manor," and other large tracts of land in this country. By his marriage with Miss Rutland, Richard Snowden became possessed of all the land within a radius of ten miles of Laurel. He started the first iron works in this region at Elk Ridge and later established those at Patuxent and Muirkirk. The Snowden family, for a long stretch of years, was among the largest landholders in Maryland, and has had, from colonial days a very numerous connection, through marriage, with the prominent families of the state.

Dr. Snowden secured a liberal education, and half a century ago was graduated in medicine from the University of Maryland. He practiced successfully until the outbreak of the war. He was among the first Marylanders to espouse the cause of the South, and became an orderly sergeant in the famous Dement Battery. Later, he was commissioned surgeon in the Second Maryland Infantry, commanded by Colonel James R. Herbert, who was a cousin of Dr. Snowden His brother, Nicholas Snowden, of the same regiment, was killed in one of the battles of Jackson's Valley campaign, in which the regiment distinguished itself. Dr. Snowden was at Gettysburg, when the regiment was annihilated and most of its field officers killed or wounded. Colonel Herbert was among the wounded. Dr. Snowden's valor and skill were often conspicuous in battle and in field hospital.

Returning to Laurel at the end of the war, impoverished but undaunted, and following Lee's advice at Appomattox, Dr. Snowden resumed the practice of his profession. He speedily built up a large and lucrative practice, and for many years was also engaged in the drug business. He amassed a competence before the weight of age led him, about four years ago, to retire. Dr. Snowden had been identified with every movement for the advancement of Laurel. He was a member of the House of Delegates from this county at the sessions of 1876 and 1880. In 1895 he was elected president of the Laurel Electric Company. He was a member of Laurel Wreath Lodge of Masons.

Dr. Snowden married Miss Emma Capron, niece of Colonel Horace Capron, the founder of Laurel. She died about twenty years ago. Dr. Snowden's immediate family consists of two sons. Messrs. John C. Snowden and De Wilton Snowden, of Laurel; and two daughters, Mrs. Emory G. Valk, of Laurel, and Mrs. Laurence Reese, of Baltimore city. Colonel R. Snowden Andrews is one of the many well-known Baltimoreans connected with Dr. Snowden's family.

